# Usefulness of wearable fitness tracking devices in patients undergoing esophagectomy

**DOI:** 10.1007/s10388-021-00893-3

**Published:** 2021-10-28

**Authors:** Junko Honke, Yoshihiro Hiramatsu, Sanshiro Kawata, Eisuke Booka, Tomohiro Matsumoto, Yoshifumi Morita, Hirotoshi Kikuchi, Kinji Kamiya, Keiko Mori, Hiroya Takeuchi

**Affiliations:** 1 Department of Perioperative Functioning Care and Support, Hamamatsu University School of Medicine, 1-20-1 Handayama, Higashi-ku, Hamamatsu, Shizuoka 431-3192 Japan; 2 Department of Surgery, Hamamatsu University School of Medicine, Hamamatsu, Japan; 3 Graduate School of Health Sciences, Okayama University, Okayama, Japan

**Keywords:** Esophagectomy, Perioperative care, Fitness trackers, Patient education as topic

## Abstract

**Background:**

Esophageal cancer surgery requires maintenance and enhancement of perioperative nutritional status and physical function to prevent postoperative complications. Therefore, awareness of the importance of preoperative patient support is increasing. This study examined the usefulness of using a diary in combination with a wearable fitness tracking device (WFT) in patients undergoing surgery for esophageal cancer.

**Methods:**

Ninety-four patients who underwent esophagectomy between February 2019 and April 2021 were included. Physicians, nurses, dietitians, and physical therapists provided diary-based education for the patients. In addition, a WFT was used by some patients. The perioperative outcomes of patients who used both the diary and WFT (WFT group) and those who used the diary alone (non-WFT group) were compared. In addition, propensity score matching was performed to improve comparability between the two groups.

**Results:**

After the propensity score matching, the rate of postoperative pneumonia was significantly lower in the WFT group (0% vs. 22.6%, *P* = 0.005). The postoperative hospital stay was shorter in the WFT group *(P* = 0.012). Nutritional status indices, such as the prognostic nutritional index, also improved significantly in the WFT group at 1 month after surgery *(P* = 0.034). The rate of diary entries was significantly higher in the WFT group (72.3% vs. 28.3%, *P* < 0.001).

**Conclusion:**

The use of a WFT reduced the incidence of postoperative pneumonia and improved postoperative nutritional status and rates of diary entries after esophagectomy, suggesting that its use may be useful for promoting recovery after esophagectomy.

**Supplementary Information:**

The online version contains supplementary material available at 10.1007/s10388-021-00893-3.

## Introduction

Despite continued advancements in minimally invasive surgery for esophageal cancer, it remains a highly invasive procedure with a high rate of postoperative complications and mortality [1–3]. Infectious complications after esophagectomy may worsen patient prognosis [4]. Previous studies have reported that sarcopenia is a risk factor for respiratory complications and anastomotic leakage after esophagectomy [5, 6]. In this regard, patients undergoing esophageal cancer surgery should have their nutritional status and physical function maintained and enhanced in the perioperative period to prevent postoperative complications. However, there are only a few reports on patient self-physical management in the perioperative period and patient education to promote recovery after surgery. Furthermore, the kind of education that would be useful remains unclear.

In 2017, the Hamamatsu Perioperative Care Team (HOPE), a multidisciplinary preoperative management team, was established [7]. Beginning in 2019, preoperative education using a treatment diary and a wearable fitness tracking device (WFT) was added to the HOPE program. The diary comprises items describing the state of the body, such as dietary intake, body weight, and exercise. The aim of keeping a diary is to help patients develop interest in their status and be willing to respond to changes in physical condition after surgery. The purpose of the WFT is to improve motivation for perioperative rehabilitation and visualize the amount of physical activity. Previous studies have reported physical activity levels using WFTs in patients with chronic diseases, such as diabetes, heart failure, and chronic obstructive pulmonary disease [8–11]. In addition, there are numerous reports on the use of WFTs in patients with cancer, including breast and colorectal cancer, showing how the use of WFT during or after treatment affects physical and psychological aspects [12, 13]. However, there are no reports on the use of WFTs for perioperative patients requiring highly invasive surgery, such as esophageal cancer surgery, and the usefulness of WFTs in the perioperative period is unclear. Therefore, we hypothesized that the preoperative use of a WFT would be associated with accelerated recovery after surgery. This study investigated the usefulness of a combination of diary and WFT in the perioperative management of esophageal cancer surgery.

## Patients and methods

### Patients

A total of 98 patients underwent esophagectomy between February 2019 and April 2021 at the Department of Surgery at Hamamatsu University School of Medicine. Of the 98 patients, 94 were included in the analysis, and 4 patients who underwent 2-stage surgery were excluded. Without preoperative treatment, HOPE intervention began when the surgery was decided after the first visit, and at the same time, the use of WFTs was confirmed. For those with neoadjuvant therapy, HOPE interventions were initiated at the time of neoadjuvant therapy initiation, and after chemotherapy, WFTs were confirmed for use when surgery was decided. Informed Consent (IC) for using WFT was obtained in all essential cases except when the device was lacking. There were 8 (8.5%) patients in whom IC was not obtained due to lack of device, 5 (5.3%) who were excluded due to inadequate understanding of WFT, 40 (42.6%) who refused to wear WFT, and 41 (43.6%) who agreed to wear WFT. Patients were excluded if they had an inadequate understanding of how to charge and operate the WFT. The reasons for refusing to wear the WFT include: (1) the patients were not used to wearing it, and (2) they felt like they were being monitored (Online Resource 1). The patients were divided into 2 groups: 41 in the WFT group and 53 in the non-WFT group.

### The HOPE protocol for esophagectomy

The HOPE protocol for esophagectomy was previously reported [7]. HOPE is composed of multiple professions that initiate interventions as soon as possible for those scheduled for surgery and provide guidance on abstinence from alcohol, tobacco use, and oral care. The patient’s physical function and nutritional status were evaluated, and the intervention was carried out according to the patient’s condition.

Following the surgery, early mobilization was encouraged while providing care to relieve pain. The dietician and rehabilitation staff regularly intervened within the first year after discharge.

### Preoperative education on using the WFT and diary

**Fig. 1:**
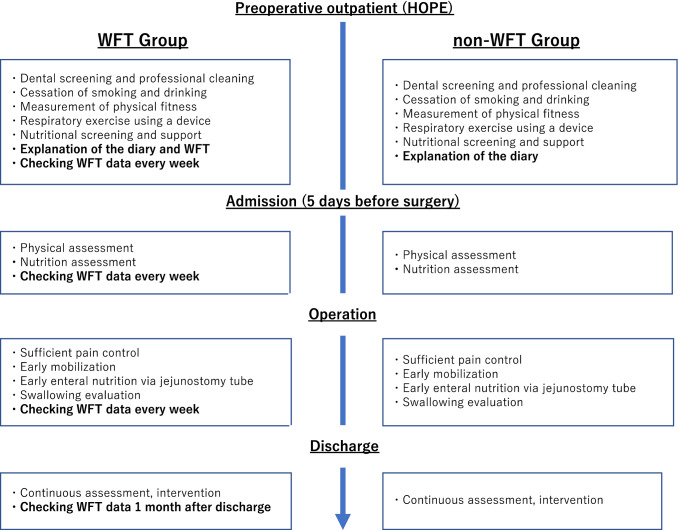
Perioperative HOPE program, including use of a diary and WFT. *HOPE* Hamamatsu Perioperative Care Team, *WFT* wearable fitness tracking device

In addition to previously instated HOPE programs for patients scheduled for esophagectomy, we explained how to use the diary and provided lifestyle guidance (Fig. 1). The patients who wore WFTs were given instructions on how to use them. The Fitbit Alta HR or inspire HR (Fitbit; San Francisco, CA) was used in this study. The Fitbit can automatically measure the patient’s heart rate, step count, calories consumed, activity time, and sleep time. In this study, the WFT and diary were used from the time of HOPE intervention until 1 month after postoperative discharge. This study was approved by the Clinical Research Ethics Committee of Hamamatsu University School of Medicine (approval No. 19–093).

### Assessment of perioperative outcomes

The duration of ventilator use and oxygen administration after extubation were summed as the length of days of postoperative oxygen administration. We assessed the occurrence of postoperative complications, including pneumonia and anastomotic leakage of grade II and higher, according to the Clavien-Dindo classification [14]. Postoperative pneumonitis was assessed using the scores on the Utrecht Pneumonia Scoring System [15]. We assessed ingestion according to the interval between surgery and oral intake initiation, which was defined as eating meals such as rice gruel and other foods, excluding fluids and jelly used for swallowing rehabilitation. In the evaluation of early mobilization, the patients were concluded to have achieved standing if they could stand the day after surgery and have achieved ambulation if they could walk a few meters using a walker. The body weight at 1 month after surgery and the weight loss rate compared with their preoperative values were calculated. Energy intake at discharge was extracted from the medical records. The average and the ratio to the nutritional requirements (NR) of energy intake for meals, oral supplementation (ONS), and enteral nutrition (EN) during the 3 days prior to discharge were calculated. NR was calculated as follows: NR = basal energy expenditure (BEE) ×1.3. BEE was calculated using the Harris-Benedict equation [16]. Nutritional status was assessed using the Glasgow prognostic score (GPS) and prognostic nutritional index (PNI), as well as serum levels of C-reactive protein (CRP), albumin, and transthyretin. PNI was calculated using two factors, blood albumin level and peripheral lymphocyte count, as reported by Onodera et al. [17]. GPS was based on a combination of CRP level and albumin level [18]. Body composition was assessed by the skeletal muscle index (SMI), body fat mass, and leg muscle mass using the Inbody S10 (Inbody, Tokyo, Japan). In addition, to assess changes in nutritional status prior to surgery, GPS was calculated at two time points: at the initial visit and prior to surgery.

Regarding use of the diary, the rate of diary entries was calculated and evaluated. When more than the minimum description, such as body weight and dietary intake, was provided, it was judged that a diary entry had been made. In this study, the WFT-wearing rate was captured from pulse recordings, and the number of days that it was worn for > 12 h/day was determined as previously described [19]. We calculated the number of days the WFT was worn as the difference between the days the WFT was worn and the preoperative days.

### Statistical analyses

Differences in continuous variables between the two groups were assessed using the Mann–Whitney *U* test, whereas nominal variables were assessed using the chi-square test or Fisher’s exact test. Patients were matched according to four variables, namely, age, sex, neoadjuvant therapy, and body mass index (BMI), to control potential confounders. Propensity scores for all patients were estimated with multiple logistic regression analysis. The two groups were identified using a 1:1 matching ratio, and no restoration extraction was performed. The caliper for matching was set to 0.03, which is the standard deviation multiplied by 0.2. Univariate analysis was performed to examine whether age, preoperative physical factors (neoadjuvant therapy, BMI, %vital capacity, forced expiratory volume % in 1 s, PNI, transthyretin level, and SMI), and WFT use were associated with postoperative pneumonia. Multiple logistic regression analysis for the association of the dependent variables with postoperative pneumonia was performed. Statistical analyses were performed using IBM SPSS statistics 27.0 for Mac (IBM, Armonk, NY, USA). The statistical significance level was set at *P* value of < 0.05.

## Results

Propensity score matching matched 31 patients in the WFT and non-WFT groups. The characteristics of patients before and after propensity score matching are shown in Table 1. Before propensity score matching, the patients in the WFT group were significantly younger and had early-stage cases in oncological factors. After propensity score matching, there were no significant differences in patient characteristics. The perioperative outcomes are shown in Table 2. There was no significant difference in the duration of postoperative oxygenation (3 vs. 4 days, *P* = 0.046) or days to initiation of oral intake (11 vs. 12 days, *P* = 0.754). Early mobilization by standing on the first day after surgery was achieved in 90.3% of patients in the WFT group and 100% in the non-WFT group, with no significant difference between the two groups (*P* = 0.119). There was also no significant difference in the percentage of patients achieving ambulation on postoperative day 1 between the WFT (58.1%) and non-WFT (64.5%) groups (*P* = 0.397). The overall postoperative complication rate of Clavien-Dindo grade II or higher was 5 (16.1%) and 12 (38.7%) (*P* = 0.043) in the WFT and non-WFT groups, respectively, with a significantly lower rate in the WFT group. The postoperative complication rate of grade III or higher was 5 (16.1%) and 11 (35.5%), respectively (*P* = 0.073). The incidence of postoperative pneumonia of Clavien-Dindo grade II or higher was significantly lower in the WFT group than in the non-WFT group (0% vs. 22.6%, *P* = 0.005). There was no significant difference in the incidence of anastomotic leakage between the two groups (WFT group 9.7% vs. non-WFT group 6.5%, *P* = 0.500). The median postoperative hospital stay was 22 days in the WFT group and 29 days in the non-WFT group, and was significantly shorter in the WFT group (*P* = 0.012). There was no significant difference between the two groups in body weight and weight loss rate at 1 month after surgery (57.3 vs. 54.7 kg, *P* = 0.226 and 5.8% vs. 5.7%, *P* = 0.569, respectively).

**Table 1:** Patient characteristics

	Before propensity score matching			After propensity score matching		
	WFT *N* = 41	Non-WFT *N* = 53	*P* value	WFT *N* = 31	Non-WFT *N* = 31	*P* value
Age (years)*	64 (60–70)	69 (65–74)	< 0.001	67 (62–71)	68 (63–73)	0.530
Sex						
Male/female	32/9	47/6	0.133	27/4	27/4	0.646
PS 0/1/2/3/4	36/5/0/0/0	37/14/1/1/0	0.185	26/5/0/0/0	21/9/0/1/0	0.263
Pathological findings						
pT 0/1/2/3/4	2/25/0/11/3	2/17/7/26/1	0.006	2/19/0/8/2	2/10/3/15/1	0.083
pN 0/1/2/3	18/14/1/8	16/17/14/6	0.014	14/10/1/6	8/11/8/4	0.057
Preoperative weight (kg)*	61.0 (53–67)	58.0 (51–64)	0.371	61.1 (56.9–66.6)	59.3 (55.5–63.2)	0.285
Body mass index (kg/m^2^)*	21.9 (20–23)	21.1 (20–25)	0.723	20.2 (19.3–22.7)	21.7 (20.4–24.0)	0.961
Brinkman index*	610(300–800)	495(0–901)	0.659	620 (400–800)	600 (158–975)	0.902
Respiratory function						
%VC (%)^†^	101.8 ± 9.3	95.8 ± 11.9	0.167	95.5 ± 20.7	98.6 ± 12.7	0.958
FEV1.0% (%)^†^	73.6 ± 7.2	72.3 ± 8.7	0.474	74.9 ± 7.6	71.9 ± 8.8	0.237
Neoadjuvant therapy	15(36.6%)	28 (52.8%)	0.087	15 (48.4%)	15 (48.4%)	0.600
Surgical approach			0.774			0.789
Thoracoscopy + laparoscopy	21 (51.2%)	25 (47.2%)		14 (45.2%)	13 (41.9%)	
Thoracoscopy + laparotomy	11 (26.8%)	15 (28.3%)		8 (25.8%)	9 (29.0%)	
Thoracotomy + laparoscopy	5 (12.2%)	7 (13.2%)		5 (16.1%)	4 (12.9%)	
Thoracotomy + laparotomy	3 (7.3%)	6 (11.3%)		3 (9.7%)	5 (16.1%)	
Mediastinoscopy + laparoscopy	1 (2.4%)			1 (3.2%)		

*WFT* wearable fitness tracking device, *PS* Performance Status, *%VC* percentage of vital capacity, *FEV1.0%* forced expiratory volume % in 1 s

*Median (interquartile range: 25th percentile to 75th percentile)

^†^Mean ± standard deviation

**Table 2:** Postoperative outcomes

	WFT *N* = 31	non-WFT *N* = 31	*P *value
Postoperative oxygenation (days)*	3 (3–4)	4 (3–8)	0.046
Initiation of oral intake (days)*	11 (10–16)	12 (9–15)	0.754
Early ambulation			
Achievement of standing^a^	28 (90.3%)	31 (100%)	0.119
Achievement of ambulation^a^	18 (58.1%)	20 (64.5%)	0.397
Any complication^b^			
Grade II or higher	5 (16.1%)	12 (38.7%)	0.043
Grade III or higher	5 (16.1%)	11 (35.5%)	0.073
Anastomotic leakage^c^	3 (9.7%)	2 (6.5%)	0.500
Pneumonia^c^	0 (0)	7 (22.6%)	0.005
Energy intake at discharge*^,d^			
Total (kcal/day)	1440 (1290–1800)	1400 (1159–1730)	0.257
Meal (kcal/day)	500 (400–790)	500 (199–637)	0.083
ONS (kcal/day)	280 (160–500)	0 (0–410)	0.049
EN (kcal/day)	600 (500–600)	600 (400–900)	0.288
Rate of energy intake at discharge*^,d,e^			
Total (%)	84.8 (69.9–99.5)	80.5 (63.7–96.4)	0.335
Meal (%)	30.5 (24.3–41.4)	27.3 (10.9–36.9)	0.107
ONS (%)	14.2 (8.6–29.5)	0.0 (0.0–22.3)	0.052
EN (%)	31.7 (28.9–37.9)	37.0 (26.8–61.2)	0.087
Postoperative hospital stay (days)*	22 (20–29)	29 (24–36)	0.012
Postoperative weight (kg)*^,f^	57.3 (52.9–62.9)	54.7 (51.7–58.6)	0.226
Rate of weight loss (%)*^,f^	5.8 (4.0–7.1)	5.7 (4.3–8.7)	0.569
Postoperative nutritional status†^,f^			
PNI	46.2 (40.8–49.7)	42.6 (37.8–45.9)	0.034
GPS 0/1/2	25/6/0	14/13/4	0.008
Albumin level (g/dl)	3.9 (3.6–4.1)	3.6 (3.2–3.9)	0.013
Transthyretin level (g/dl)	24.4 (21.5–26.0)	19.4 (15.0–22.9)	0.001
CRP level (mg/dl)	0.14 (0.09–0.31)	0.35 (0.15–1.60)	0.018
Postoperative body composition*^,f^			
SMI (kg/m^2^)	7.0 (6.2–7.3)	6.3 (6.2–7.2)	0.228
Body fat (%)	21.0 (16.3–23.9)	21.7 (17.6–27.3)	0.416

*WFT* wearable fitness tracking device, *ONS* oral nutrition supplementation, *EN* enteral nutrition, *PNI* prognostic nutritional index, *GPS* Glasgow prognostic score, *CRP* C-reactive protein, *SMI* skeletal muscle mass index

*Median (interquartile range: 25th percentile to 75th percentile)

^†^Mean ± standard deviation

^a^Achieved on postoperative day 1

^b^Clavien-Dindo grade Classification

^c^Clavien-Dindo grade II or higher

^d^Average of 3 days before discharge

^e^Rate of intake relative to nutritional requirements

^f^Values at 1 month after surgery rate of change in the values from preoperative to 1 month postoperative

The energy intake from food and ONS at discharge was significantly increased in the WFT group (500 vs. 500 kcal, *P* = 0.083 and 280 vs. 0 kcal, *P* = 0.049, respectively). There was no significant difference in energy intake from EN between the two groups (600 vs. 600 kcal, *P* = 0.288). There was also no significant difference in the proportions of food intake, ONS, and EN between the two groups (30.5% vs. 27.3%, *P* = 0.107; 14.2% vs. 0%, *P* = 0.052; 31.7% vs. 37.0%, *P* = 0.087, respectively). The preoperative PNI was not significantly different between the two groups in terms of nutritional assessment. However, at 1 postoperative month, the PNI was significantly better in the WFT group than in the non-WFT group (46.2 vs. 42.6, *P* = 0.034). Serum albumin and transthyretin levels were also significantly higher in the WFT group (3.9 vs. 3.6 g/dl, *P* = 0.013 and 24.4 vs. 19.4 g/dl, *P* = 0.001, respectively). CRP levels were significantly lower in the WFT group (0.14 vs. 0.35 mg/dl, *P* = 0.018). SMI and body fat were not significantly different between the two groups (7.0 vs. 6.3 kg/m^2^, *P* = 0.228 and 21.0% vs. 21.7%, *P* = 0.416, respectively). Similar results were observed in GPS (Table 2, Online Resource 2). There was no significant difference in perioperative GPS between the two groups at the initial visit (GPS 0/1/2: 26/5/0 vs. 24/5/2, *p* = 0.353). Just before surgery, GPS was improved in the WFT group (score 0/1/2: 31/0/0 vs. 26/4/1, *P* = 0.066), and was also significantly improved in the WFT group 1 month after surgery (score 0/1/2: 25/6/0 vs. 14/13/4, *P* = 0.008).

Multiple logistic regression analysis revealed that the use of WFT significantly reduced the incidence of postoperative pneumonia (odds ratio [OR] 0.086, confidence interval [CI] 1.31–102.23, *P* = 0.027; Table 3).

**Table 3:** Logistic regression analysis of postoperative pneumonia

	Univariate logistic regression (*N* = 94)			Multiple logistic regression (*N* = 94)		
	OR	*P* value	95% CI	OR	*P* value	95% CI
Age (years)	1.037	0.366	0.96–1.12	1.03	0.468	0.95–1.12
Neoadjuvant therapy (yes/no)	0.670	0.535	0.19–2.37	0.80	0.748	0.20–3.15
Preoperative body mass index (kg/m^2^)	0.968	0.754	0.79–1.19			
Preoperative %VC (%)	0.993	0.730	0.96–1.03			
Preoperative FEV1.0% (%)	0.978	0.572	0.91–1.06			
Preoperative PNI	1.015	0.804	0.90–1.14			
Preoperative transthyretin level (g/dl)	1.035	0.522	0.93–1.15			
Preoperative SMI (kg/m^2^)	1.273	0.484	0.689–2.36	1.75	0.151	0.81–3.78
Use of WFT (yes/no)	0.111	0.037	1.14–75.98	0.086	0.027	1.31–102.23

*OR* odds ratio, *95% CI* confidence interval, *%VC* percentage of vital capacity, *FEV1.0%* forced expiratory volume % in 1 s, *PNI* prognostic nutritional index, *SMI* skeletal muscle mass index, *WFT* wearable fitness tracking device

The outcomes associated with the diary are shown in Table 4. The diary entry rate was significantly higher in the WFT group (72.3% vs. 28.3%, *P* < 0.001). The majority of patients in the WFT group had diary entry rates of ≥ 70%, while the majority in the non-WFT group had ≤ 50%. There was one patient in the WFT group and two in the non-WFT group who lost their diary, but the difference was not significant between the two groups (1/30 [3.3%] vs. 2/21 [9.5%], *P* = 0.361).

**Table 4:** Diary outcomes

	WFT *N* = 30	Non-WFT *N* = 21	*P* value
Rate of description (%)*	72.3 ± 31.6	28.3 ± 12.4	< 0.001
≥ 50% of the description	23 (76.7%)	2 (9.5%)	< 0.001
Loss of diary	1 (3.3%)	2 (9.5%)	0.361

*WFT* wearable fitness tracking device

*Mean ± standard deviation

The mean WFT-wearing rate was 91.8% ± 11.8%, and it was more than 80% in 35 cases (85.4%) (Online Resource 3). The mean preoperative step count in patients with WFT was 7662 ± 3066 steps/days. When the mean preoperative step count was divided into two groups (≥ 7000 and < 7000 steps/day), there was no significant difference in perioperative outcomes. However, the oral intake (meal) rate at discharge (35.6% vs. 24.2%, *P* = 0.040) and diary entries (84.7% vs. 64.0%, *P* = 0.043) were also higher in the group that walked ≥ 7000 steps/day preoperatively compared with the other group (Online Resource 4–6).

## Discussion

The main findings of this study were that patients wearing a WFT had a significantly lower rate of postoperative pneumonia. In addition, the use of WFTs was found to increase the rate of diary entries and affect the postoperative course. To our knowledge, this is the first study to report that WFT promotes early recovery after esophagectomy.

Preoperative education for patients undergoing highly invasive surgery, such as esophageal cancer resection, is particularly critical to support patients in anticipation of early postoperative recovery. Previous studies on WFTs and step counts have reported that daily checks may lead to greater awareness of measured and targeted values and also increase step counts [20–22]. The amount of physical activity before esophagectomy is associated with the risk of postoperative pneumonia, and the incidence of pneumonia decreases as the amount of physical activity increases [23]. In this study, the incidence of postoperative pneumonia was lower in the WFT group than in the non-WFT group, suggesting that the use of WFT may have maintained and increased physical activity before surgery. Although this is indirect evidence because the amount of activity in the non-WFT group was not visualized, GPS was improved preoperatively in the WFT group. This suggests that the nutritional status of the WFT group may have improved along with maintenance and increase in activity, which may have consequently contributed to reductions in postoperative pneumonia.

The incidence of pneumonia (22.6%) in the non-WFT group of this study was slightly high, but it was not considered to be remarkably higher than approximately 15% of postoperative pneumonia, according to the analysis of the Japanese National Clinical Database [1]. Additionally, pneumonia may be diagnosed more often in our facility than in other facilities because the computed tomography is routinely examined in the first week postoperatively in our facility.

WFTs have been reported to be effective in promoting activity after surgery. A randomized controlled trial (RCT) of colorectal and endometrial cancer survivors who had completed active cancer treatment showed that the physical activity was significantly improved in the intervention group [12]. A pilot RCT of non-metastatic or recurrent colorectal cancer patients who had completed curative therapy showed that the WFT increased their motivation to exercise [24]. Although these studies used post-treatment interventions, the use of WFT before surgery, as in this study, can similarly motivate physical activity and promote recovery, which may influence perioperative outcomes. Postoperative pneumonia is a common complication after esophagectomy. For the purpose of reducing postoperative complications such as pneumonia, it may be useful to visualize preoperative physical activity of the patients as much as possible and voluntarily increase their activity using WFT.

Postoperative EN might decrease the incidence of pneumonia after esophagectomy [25], and all patients in this study received EN postoperatively. Moreover, it is also recommended that oral intake be prioritized over EN and parenteral nutrition in postoperative nutritional management [26]. The WFT group had shorter postoperative hospital stays and better PNI and albumin levels at 1 month after surgery, suggesting that oral intake was stabilized earlier than the non-WFT group. One of the reasons for the better oral intake in the WFT group was that the patients using WFT might have been able to maintain their motivation for physical activity after the surgery and restore their oral intake according to their activity level. In the WFT group, patients with a mean preoperative step count of ≥ 7000 steps/day tended to have a higher meal intake than those with a mean preoperative step count of ≤ 7000 steps/day, suggesting that the activity and oral intake are related. The non-WFT group had a higher incidence of postoperative pneumonia than the WFT group, and the prolonged inflammation may have led to reduced oral intake. Although we did not investigate the dietary intake at 1 month postoperatively, these findings suggest that oral intake in the WFT group may be higher at 1 month.

This study showed that the rate of diary entries was higher in the WFT group. Objective monitoring of the patients’ physical condition using the WFT may have encouraged them to maintain diary entries. To produce behavioral changes in daily life, patients require feedback on their behaviors [27, 28]. We assumed that the diary would be the patient’s own self-assessment tool and that this feedback would motivate their preparations for surgery. The greater attention paid to diary maintenance in patients using WFTs may have motivated them to recover after surgery. In this study, the rate of diary entries was low in the non-WFT group that used diary only, and the rate of diary entries was high when used in combination with WFTs. Therefore, it is considered that the effect of the diary alone is limited to the subjects of this study. A previous RCT of patients with heart failure using diaries found that higher diary use leads to longer survivorship, suggesting that higher engagement in self-care behaviors is associated with improvement of heart failure outcomes [29]. The present study also showed a high rate of diary entries with the WFT, which may have led to increasing involvement in self-care behaviors and usefulness of the diary. This appeared to have affected the maintenance of physical activity, early stabilization of oral intake, and early discharge after surgery.

This study has some limitations. First, the WFT group comprised patients who voluntarily used WFTs. Thus, patients in the WFT group may have been more invested in managing their bodies than those in the non-WFT group. The use of WFT as indicated may be influenced by the patient's willingness to participate in the treatment. However, we believe that not only patient’s willingness to be treated, but also various factors influenced their participation in this study, such as non-participation due to lack of WFT in the initial period of the study and refusal to use devices due to patients’ daily habits. It is difficult to visualize and quantify factors, such as patients’ willingness to be treated and habits; therefore, it is impossible to add to the adjustment factors for propensity score matching, and we believe that this is one of the key limitations of this study. Second, there is no objective data to show the amount of activity in the non-WFT group. Therefore, it is not possible to make a direct comparison with the WFT group regarding preoperative activity. In this study, we estimated activity in the non-WFT group based on preoperative nutritional status. In addition, the interval between the initial visit and surgery varied from patient to patient, and these data are preliminary and considered as limitation to the study.

Third, the results of this study may not be universal, as only a limited number of cases from a single center are included in the analysis. However, this study is the first attempt to analyze the usefulness of the WFT in perioperative patients with esophageal cancer. It is also expected that mobile devices will be widely operationalized in the future healthcare fields and the prospects that the available information will be obtained [30]. Mobile devices are also expected to be applied in the surgical field, and we believe that this study will provide foundational material for future multi-institutional collaborations and studies with a high level of evidence.

Future directions for this area of research include increasing the number of cases and verifying the effective interventions and long-term effects of patient support using a WFT and diary prior to esophagectomy.

## Conclusion

In this study, the use of WFT was associated with reduced incidence of postoperative pneumonia, early stabilization of oral intake, and improved rates of diary maintenance. These findings suggest that the use of WFT can help promote early recovery after esophagectomy.

## Supplementary Information

Below is the link to the electronic supplementary material.

Supplementary file1 (PDF 154 kb)

Supplementary file2 (PDF 156 kb)

Supplementary file3 (PDF 196 kb)

Supplementary file4 (PDF 183 kb)

Supplementary file5 (PDF 188 kb)

Supplementary file6 (PDF 160 kb)
