# Supplementary material for: Usefulness of wearable fitness tracking devices in patients undergoing esophagectomy
Source: Esophagus. 2021 Oct 28;19(2):260–8. doi: 10.1007/s10388-021-00893-3 (PMC8921159; doi:10.1007/s10388-021-00893-3)
Supplement: Supplementary file 4 — Supplementary file4 (PDF 183 kb) [file 10388_2021_893_MOESM4_ESM.pdf]

Online Resource 4. Patient characteristics of the groups divided by preoperative step counts

	Preoperative step counts		
	≥7000 steps/day<br>$N = 25$	<7000 steps/day<br>$N = 16$	P-value
Age (years)*	63 (60-70)	68 (61-70)	0.721
Sex			
Male/female	17/8	15/1	0.055
PS 0/1/2/3/4	22/3/0/0/0	14/2/0/0/0	0.659
Pathological findings			
pT 0/1/2/3/4	1/17/7/0	1/8/4/3	0.149
pN 0/1/2/3	13/14/1/8	5/6/0/5	0.316
Preoperative weight (kg)*	61.1 (49.6-66.7)	60.1 (57.3-66.9)	0.682
Body mass index (kg/m^2)*	21.8 (19.8-23.1)	21.9 (20.2-23.4)	0.885
Brinkman index*	600(0-800)	615(400-719)	0.702
Respiratory function			
%VC (%) †	97.1 ± 22.4	98.9 ± 13.7	0.988
FEV1.0% (%) †	75.0 ± 7.4	73.9 ± 6.8	0.510
Neoadjuvant therapy	8 (32.0%)	7 (43.8%)	0.332
Surgical approach			0.075
Thoracoscopy + laparoscopy	16 (64.0%)	5 (31.3%)	
Thoracoscopy + laparotomy	5 (20.0%)	6 (37.5%)	
Thoracotomy + laparoscopy	3 (12.0%)	2 (12.5%)	
Thoracotomy + laparotomy	0 (0)	3 (18.8%)	
Mediastinoscopy＋laparoscopy	1 (4.0%)		

WFT, wearable fitness tracking device; PS, Performance Status; %VC, percentage of vital capacity; FEV1.0%, forced expiratory volume % in 1 s.

*Median (interquartile range: 25th percentile to 75th percentile)

†Mean ± standard deviation

Title: Usefulness of wearable fitness tracking devices in patients undergoing esophagectomy

Journal name: Esophagus

Junko Honke, RN, MSN[1]; Yoshihiro Hiramatsu, MD, PhD[1,2]; Sanshiro Kawata, MD, PhD[2]; Eisuke Booka, MD, PhD[2]; Tomohiro Matsumoto, MD[2]; Yoshifumi Morita, MD, PhD[2]; Hirotoshi Kikuchi, MD, PhD[2]; Kinji Kamiya, MD, PhD[2]; Keiko Mori, RN, PhD[3]; Hiroya Takeuchi, MD, PhD[2]

[1]Department of Perioperative Functioning Care and Support, Hamamatsu University School of Medicine, 1-20-1 Handayama, Higashi-ku, Hamamatsu 431-3192, Japan

[2]Department of Surgery, Hamamatsu University School of Medicine, Hamamatsu, Japan

[3]Graduate School of Health Sciences, Okayama University, Okayama, Japan

Corresponding author: Yoshihiro Hiramatsu, MD, PhD.

E-mail: hiramatu@hama-med.ac.jp
