# Supplementary material for: Usefulness of wearable fitness tracking devices in patients undergoing esophagectomy
Source: Esophagus. 2021 Oct 28;19(2):260–8. doi: 10.1007/s10388-021-00893-3 (PMC8921159; doi:10.1007/s10388-021-00893-3)
Supplement: Supplementary file 5 — Supplementary file5 (PDF 188 kb) [file 10388_2021_893_MOESM5_ESM.pdf]

Online Resource 5. Postoperative outcomes of the groups divided by preoperative step counts

	Preoperative step counts		
	≥7000 steps/day<br>*N* = 25	<7000 steps/day<br>*N* = 16	*P*-value
Postoperative oxygenation (days)*	3 (2-4)	4 (3-5)	0.095
Initiation of oral intake (days)*	10 (9-15)	11 (10-13)	0.259
Early ambulation			
Achievement of standing[1]	23 (92.0%)	15 (93.8%)	0.666
Achievement of ambulation[1]	15 (60.0%)	9 (56.3%)	0.533
Any complication			
Grade II	3 (12.0%)	3 (18.8%)	0.400
Grade III	5 (20.0%)	2 (12.5%)	0.431
Anastomotic leakage[2]	2 (8.0%)	1 (6.3%)	0.666
Pneumonia[2]	0 (0)	1 (6.3%)	0.390
Energy intake at discharge*,[3]			
Total (kcal/day)	1440 (1280-1800)	1550 (1235-1825)	0.802
Meal (kcal/day)	630 (500-795)	420 (300-765)	0.085
ONS (kcal/day)	300 (0-400)	400 (200-400)	0.552
EN (kcal/day)	600 (600-600)	600 (550-900)	0.154
Rate of energy intake at discharge*,[3,4]			
Total (%)	84.1 (71.5-99.4)	84.2 (68.0-94.3)	0.702
Meal (%)	35.6 (27.3-49.3)	24.2 (16.9-41.3)	0.040
ONS (%)	14.7 (0-27.3)	18.8 (11.0-21.0)	0.702
EN (%)	31.7 (27.6-35.6)	32.3 (28.4-45.2)	0.361
Postoperative hospital stay (days)*	23 (20-26)	22 (20-28)	0.989
Postoperative weight (kg)*,[5]	57.3 (47.8-61.6)	56.9 (54.7-63.4)	0.625
Rate of weight loss (%)*,[6]	5.4 (4.0-6.8)	5.3 (2.3-7.1)	0.552
Postoperative nutritional status†,[5]			
PNI	45.8 (42.5-48.5)	47.3 (41.1-53.8)	0.251
GPS 0/1/2	22/3/0	13/3/0	0.434
Albumin level (g/dl)	3.9 (3.6-4.1)	4.0 (3.6-4.1)	0.864
Transthyretin level (g/dl)	23.9 (21.4-25.4)	23.2 (19.2-26.2)	0.499
CRP level (mg/dl)	0.13 (0.09-0.25)	0.25 (0.12-0.41)	0.095
Postoperative body composition*,[5]			
SMI (kg/m^2)	7.1 (5.4-7.4)	6.6 (6.3-7.1)	0.823
Body fat (%)	22.2 (19.0-23.9)	20.4 (15.5-23.0)	0.346

WFT, wearable fitness tracking device; ONS, oral nutrition supplementation; EN, enteral nutrition; PNI, prognostic nutritional index; GPS, Glasgow prognostic score; CRP, C-reactive protein; SMI, skeletal muscle mass index.

*Median (interquartile range: 25th percentile to 75th percentile)

†Mean ± standard deviation

[1]Achieved on postoperative day 1

[2]Clavien-Dindo grade II or higher

[3]Average of 3 days before discharge

[4]Rate of intake relative to nutritional requirements

[5]Values at 1 month after surgery

[6]Rate of change in the values from preoperative to 1 month postoperative

Title: Usefulness of wearable fitness tracking devices in patients undergoing esophagectomy

Journal name: Esophagus

Junko Honke, RN, MSN[1]; Yoshihiro Hiramatsu, MD, PhD[1,2]; Sanshiro Kawata, MD, PhD[2]; Eisuke Booka, MD, PhD[2]; Tomohiro Matsumoto, MD[2]; Yoshifumi Morita, MD, PhD[2]; Hirotoshi Kikuchi, MD, PhD[2]; Kinji Kamiya, MD, PhD[2]; Keiko Mori, RN, PhD[3]; Hiroya Takeuchi, MD, PhD[2]

[1]Department of Perioperative Functioning Care and Support, Hamamatsu University School of Medicine, 1-20-1 Handayama, Higashi-ku, Hamamatsu 431-3192, Japan

[2]Department of Surgery, Hamamatsu University School of Medicine, Hamamatsu, Japan

[3]Graduate School of Health Sciences, Okayama University, Okayama, Japan

Corresponding author: Yoshihiro Hiramatsu, MD, PhD.

E-mail: hiramatu@hama-med.ac.jp
