# Supplementary material for: Usefulness of wearable fitness tracking devices in patients undergoing esophagectomy
Source: Esophagus. 2021 Oct 28;19(2):260–8. doi: 10.1007/s10388-021-00893-3 (PMC8921159; doi:10.1007/s10388-021-00893-3)
Supplement: Supplementary file 6 — Supplementary file6 (PDF 160 kb) [file 10388_2021_893_MOESM6_ESM.pdf]

Online Resource 6. Diary outcomes of the groups divided by preoperative step counts

	Preoperative step counts		
	≥7000 steps/day<br>$N = 24$	<7000 steps/day<br>$N = 14$	P-value
Rate of description (%) *	84.7 ± 23.1	64.0 ± 31.9	0.043
≥50% of the description	23 (95.8%)	8 (57.1%)	0.006
Loss of diary	1 (4.3%)	0 (0)	0.632

WFT, wearable fitness tracking device.

*Mean ± standard deviation

Title: Usefulness of wearable fitness tracking devices in patients undergoing esophagectomy

Journal name: Esophagus

Junko Honke, RN, MSN[1]; Yoshihiro Hiramatsu, MD, PhD[1,2]; Sanshiro Kawata, MD, PhD[2]; Eisuke Booka, MD, PhD[2]; Tomohiro Matsumoto, MD[2]; Yoshifumi Morita, MD, PhD[2]; Hirotoshi Kikuchi, MD, PhD[2]; Kinji Kamiya, MD, PhD[2]; Keiko Mori, RN, PhD[3]; Hiroya Takeuchi, MD, PhD[2]

[1]Department of Perioperative Functioning Care and Support, Hamamatsu University School of Medicine, 1-20-1 Handayama, Higashi-ku, Hamamatsu 431-3192, Japan

[2]Department of Surgery, Hamamatsu University School of Medicine, Hamamatsu, Japan

[3]Graduate School of Health Sciences, Okayama University, Okayama, Japan

Corresponding author: Yoshihiro Hiramatsu, MD, PhD.

E-mail: hiramatu@hama-med.ac.jp
